# Cytotoxic and apoptotic potential of *Phyllodium elegans* extracts on human cancer cell lines

**DOI:** 10.1080/21655979.2019.1682110

**Published:** 2019-10-29

**Authors:** Seunghwa Jung, Juhyun Shin, Jeongheon Oh, Gansukh Enkhtaivan, Sang Woo Lee, Judy Gopal, Kongmany Sydara, Ramesh Kumar Saini, Young-Soo Keum, Jae-Wook Oh

**Affiliations:** a Department of Stem Cell and Regenerative Biotechnology, KIT, Seoul, Korea; b Department of Bioresource and Food Science, Konkuk University, Seoul, Korea; c International Biological Material Research Center, Korea Research Institute of Bioscience and Biotechnology, Daejeon, Republic of Korea; d Ministry of Health, Institute of Traditional Medicine, Vientiane, Lao PDR; e Department of Crop Science, Konkuk University, Seoul, Korea

**Keywords:** *Phyllodium elegans*, alcohol extract, apoptosis, Mu-2-related death-inducing gene, mitochondrial membrane potential

## Abstract

The extract of *Phyllodium (P.) elegans* was investigated for its anti-cancer properties on brain astroglioma cells (U251-MG), colorectal carcinoma cells (HCT116), and malignant melanoma cells (A375). *P. elegans* methanolic extract (PeME) showed cytotoxicity on all three cancer cell lines tested. The cell viability assay revealed that PeME significantly reduced the viability of these cells. Clear apoptotic features such as cellular morphology, cell shrinkage, and augmentation of dead cells were observed. Flow cytometry and fluorescence staining techniques confirmed the apoptotic property of PeME. *In vitro* scratch invasion assay showed that cell migration rate was significantly reduced. Fluorescence microscopic studies using 4′,6-diamidino-2-phenylindole staining showed early and late signs of apoptosis after PeME treatment. Upon PeME stimulation, activation of caspase-3/-9 and Mu-2-related death-inducing gene (MUDENG, MuD) was observed by western blot analysis. JC-1 staining analysis by flow cytometry showed that PeME depolarized the mitochondria membrane potential (MMP). Collectively, these findings, for the first time, point to the fact that PeME has anti-cancer properties against brain, colon, and skin cancer cell lines by depolarizing the MMP and activating apoptotic signaling through the activation of caspase-3/-9 as well as MuD. This is the first report reporting the anticancer activity of this specific plant extract.

**Figure:**
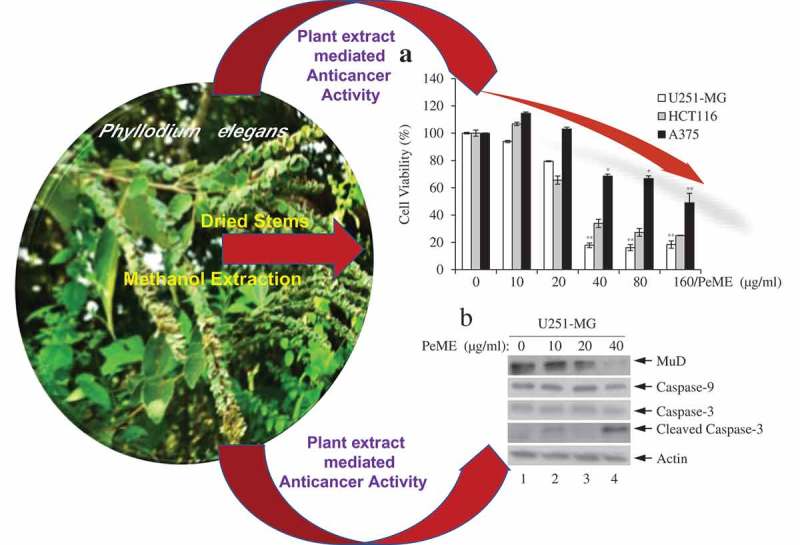

## Introduction

In recent years, remarkable progress has been made toward the understanding of cancer and its treatment. However, in spite of improved state-of-the-art-medicines such as radiation therapy, surgery, and chemotherapy, the survival rate and life expectancy of cancer patients have not been extended owing to side effects and resistance to anti-cancer drugs [1,2]. Therefore, nature-based or nature-derived medicines are receiving increasing attention as better therapeutic options for the prevention of human cancers [3,4]. To date, a large part of chemotherapeutic drugs originates from naturally-derived products [5]. The traditional use of plants for medicinal purposes has attracted extensive research attention to explore possible bioactive components for anti-cancer drugs [6,7]. Furthermore, numerous plant products are known to induce apoptosis in cancer cells but not in normal cells [8].

*Phyllodium (P)* is a small genus within the tribe Desmodieae (Leguminosae-Papilionoideae) that is distributed in India, southeastern and eastern Asia, and northern Australia [9]. *P. elegans* (Lour) Desv. (Taxon Name) is one of the six *Phyllodium* species, referred to as *Desmodium blandum, D. elegans, Dicerma elegans, Hedysarum elegans* as synonyms [10]. The roots and leaves are used medicinally to reduce fever and as an antiphlogistic, carminative, and diuretic in humans [10–12]. According to the Editorial Committee of Zhonghua Bencao National Traditional Chinese Herb Administration [13], a paste of the whole plant is also employed to stop bleeding and in the treatment of burn injuries, fractures, and bruises [14]. The root is used in the treatment of rheumatic pain and chronic hepatitis [14].

Apoptosis is a pivotal mode of action through intrinsic or extrinsic pathways by anti-tumor agents. In cells undergoing apoptosis, a family of proteases called caspases is activated. Activation of caspases appears to be directly responsible for many molecular and structural changes in the apoptotic pathway [15,16].

Mu-2-related death-inducing gene (MUDENG, MuD) protein contains a Mu homology domain found in adaptor proteins that play important roles in intracellular trafficking pathways. It is known to be involved in the initial stages of cell death in cytotoxic T cells [17]. Furthermore, we have reported that MuD possesses anti-apoptotic functions [18], and is involved in silver nanoparticle (AgNP)-induced astroglioma hormesis [19]. In addition, we reported the correlation between graviola anti-cancer activity and MuD [20].

Currently, no published reports exist reporting the anti-cancer activity of *P. elegans* (Lour.) Desv. Thus, this study was performed to investigate the cytotoxic activity of *P. elegans* methanolic extract (PeME) against three human cancer cell lines. This is the first time report that PeME treatment-induced cell viability reduction, morphological alteration, and cellular apoptosis through alleviation of mitochondrial membrane potential (MMP) on brain astroglioma cells, colorectal carcinoma cells, and human malignant melanoma cells. This less studied extract adds novel value in natural product research and is expected to trigger off sensitization for application toward versatile anticancer natural remedies.

## Materials and methods

### Chemicals and reagents

FITC-Annexin V and propidium iodide (PI) were bought from BD Biosciences (San Jose, CA, USA). JC-1 dye was purchased from Enzo Life Sciences (Farmingdale, NY, USA). 4′,6-diamidino-2-phenylindole (DAPI) was purchased from Sigma-Aldrich (Saint Louis, MO, USA). Primary antibody (Ab) for caspase-3 was from Cell Signaling Technology (Beverly, MA, USA). Caspase-9 Ab and actin Ab were from Santa Cruz Biotechnology (Dallas, TX, USA). Mouse MuD monoclonal Abs (MAb) were produced from C22B3 hybridoma culture medium [21]. Horseradish peroxidase (HRP)-conjugated goat anti-mouse IgG Ab and goat anti-rabbit IgG Ab were purchased by the Jackson ImmunoResearch Lab (West Groove, PA, USA). 2-(4-iodophenyl)-3-(4-nitrophenyl)-5-(2,4-disulfophenyl)-2H-tetrazolium monosodium salt (WST-1) reagent was purchased from Roche Applied Science (Mannheim, Germany). Recombinant Human sTRAIL/Apo2 ligand was purchased from PeproTech (Seoul, Korea).

### Preparation of P. elegans extract

Methanol extract of *P. elegans* stem was supplied by Foreign Plant Extract Bank (no. FBM118-023; Daejeon, Korea). The plant was collected by Sydara K (one of the authors of the paper) in Ham Ao, Laos in 2010 and authenticated by Institute of Traditional Medicine (ITM). A voucher specimen recoded as ‘Korea Research Institute of Bioscience and Biotechnology (KRIBB) 0033580ʹ was deposited in the herbarium of the Korea KRIBB. Briefly, the dried and refined aerial parts of *P. elegans* (92g) were extracted with 1,000 mL of 99.9% (v/v) methanol using a sonicator (SDN-900H, SD Ultrasonic Cleaner, Seoul, Korea) at 45°C for 3 days (15 min sonication at 1500 W and 40 kHz followed by 2 h standing; repeated 10 times per day). The resultant product was filtered with non-fluorescent cotton, condensed using a rotary evaporator (N-1000SWD, EYELA, Tokyo, Japan) under reduced pressure at 45°C, and lyophilized using a freeze dryer (Christ, Germany). Dimethyl sulfoxide (DMSO) was used as a solvent for lyophilized substances.

### Liquid chromatography (LC)- mass spectrometry (MS) analysis of the methanolic extract

LC- electrospray ionization (ESI)-MS, LC- ESI-tandem mass spectrometric (MS/MS), and LC-ESI-MS/MS-multiple reaction monitoring (MRM) studies were performed for the identification of bioactive compounds in the methanolic extract of *P. elegans*. In the first step, methanolic extract was partitioned with ethyl acetate to minimize the background noise in the MS analysis. Briefly, 5 mg of isolated extract was dissolved in 3 ml water and partitioned with 5 ml ethyl acetate. The upper ethyl acetate fraction was collected the procedure was repeated three times. Subsequently, all the fractions were pooled, dried in a rotary vacuum evaporator, recovered in 1 ml methanol, and filtered through a Whatman (0.45 µm) filter.

The ESI-MS and MS/MS analyses were performed by SCIEX API 3200 triple quadrupole mass spectrometer (AB-SCIEX, USA) equipped with an Exion LC™ system and Turbo V™ interface with Turbo spray ion source. The ESI-MS and MS/MS analysis were performed in negative (-) mode, following the liquid chromatographic (LC)-separation in Luna® 5 µm C18(2) column (100 Å, 250 × 4.6 mm) with TMS end-capping. The column thermostat was maintained at 20°C. The solvent system consisting of 0.1% formic acid (v/v) in water (mobile phase A) and acetonitrile with 0.1% formic acid (Mobile phase B). The gradient elution was 10–50% B in 30 min, 50–100% B in next 15 min, hold at 100% B for 10 min, followed by 5-minute post-run at a flow rate of 1 ml/min.

The ESI-MS source parameters were optimized as follows: dry gas, N_2_; curtain gas (CUR), 30 psi; ion source gas (GAS1), 50 psi; GAS2, 50 psi; temperature, 700°C; and ion spray voltage, 4000 V. The Q1 scan was performed at delustring potential (DP) of −25 to −50 V and the mass spectra were acquired in the range of 60–1000 m/z at 1 s interval.

The LC-MS/MS-MRM based targeted metabolomics approach was employed for the identification of commonly occurring polyphenols in the extract. The optimized values of collision energy [22], cell exit potential (CXP), decluttering potential (DP), entrance potential [16] of selected MRM transition that were used for identification are listed in Table S1 (supplementary material).

### Preparation of cell line and cell culture for testings

Human malignant astroglioma cell line U251-MG was obtained from Dr Benveniste EN (University of Alabama at Birmingham, Birmingham, AL, USA). Human colorectal carcinoma cell line HCT116 and human malignant melanoma cell line A375 were obtained from Korean Cell Line Bank (Seoul, Korea). U251-MG were cultured in Minimum Essential Medium (MEM) (Welgene, Daegu, Korea), HCT116 in McCoy’s 5A Medium (McCoy’s) (Welgene), and A375 cells in Dulbecco’s Modified Eagle Medium (DMEM) (Welgene). Media were supplemented with 10% (v/v) fetal bovine serum (FBS) (Gibco, Logan, Utah, USA), 1x antibiotic (Welgene).

### Cell viability assay

2.0 × 10^4^ cells were plated in a 96-well microplate and incubated overnight. The next day, PeME-containing media of specified concentrations were replenished in triplicates. After 24 h, cells were washed with phosphate buffer saline (PBS). Then, 10 µl WST solution (0.5 mg/ml) was added in each well and incubated for an additional hour in the dark. Cell viability was measured using a colorimetric assay with WST-1 reagent. The absorbance was determined at 450 nm using a microplate reader and read with Gen5 software (Bio-Tek, Winooski, VT, USA). Methanol in medium was used as a control.

### Morphological observation

2 × 10^4^ cells were plated in a six-well plate and incubated overnight. The next day, the cells were treated with PeME of specified concentration, and cellular morphological changes were examined and imaged at 24 h under an inverted phase-contrast microscope (Motic, Kowloon, Hong Kong).

### In vitro scratch motility assay

2 × 10^5^ cells were plated in a six-well plate and incubated overnight. A liner scratch wound was created after 24 h in 1% serum-containing medium with the help of a sterile 0.2 ml yellow tip. Cell debris was removed by PBS wash (done twice), followed by PeME treatment. The wound was analyzed and photographed at 12, 24, and 36 h under an inverted phase-contrast microscope and imaged by Motic Image Plus 2.0 ML (Motic).

### Nuclear morphology study by 4′,6-diamidino-2-phenylindole (DAPI) staining

2 × 10^4^ cells were seeded in a 96-well plate and incubated overnight. Cells were treated with a specified concentration of PeME. After 24 h, cells were fixed with 4% paraformaldehyde for 20 min and permeabilized with PBS containing 0.1% Tween 20 for 10 min. After washing by PBS, the cells were treated with 300 nM DAPI solution for 5 min. Immediately, the cells were analyzed under a fluorescence microscope and imaged using NIS-Elements BR (Nikon, Melville, NY, USA).

### Immunoblotting assay

Prepared cells were washed in PBS and lysed in lysis buffer as shown previously [21]. Protein concentrations were determined using the Lowry protein assay (Bio-Rad, Hercules, CA) and quantified by Microplate Spectrophotometer (Bio-tek) at 750 nm. Total proteins in each cell lysate were resolved at 10% concentration of sodium dodecyl sulfate-polyacrylamide gel electrophoresis (SDS-PAGE) and transferred onto a polyvinyl difluoride membrane. After blocking for 1 h by 5% nonfat milk at 21‒23℃, membranes were incubated with shaking overnight with specific Ab at 4°C. After incubating, PVDF membranes were carried out by incubation with HRP-conjugated secondary Ab at 21‒23°C for 2 h. Bands were visualized by enhanced chemiluminescence (Bio-Rad).

### Flow cytometric analysis

Each cell was seeded into six-well plates at 2 × 10^5^ cells/well and incubated overnight. PeME was treated into each well and incubated for 24 h. Then, the cells were washed twice and resuspended in 1 ml of 1x Annexin V binding buffer at a concentration of 1 × 10^6 ^cell/ml. Following this, 100 µl of the resuspended solution (1 × 10^5^ cells) was transferred into a 1.5 ml microtube and 5 µl of FITC-Annexin V and 5 µl of PI were added. After incubation for 20 min at 21‒23°C in the dark, 400 µl of 1x Annexin V binding buffer was added to each sample. Then, cells were analyzed by flow cytometry using NovoCyte 1000 and visualized by NovoExpress (ACEA Biosciences, San Diego, CA, USA).

### Measurement of mitochondria membrane potential (MMP)

2 × 10^5^ cells/well were seeded in a six-well plate and incubated overnight. The next day, various concentrations of PeME were treated for 24 h, and then cells were harvested with trypsin-EDTA (Welgene) and transferred into 1.5 ml microtubes (1 × 10^4^ cell/tube). JC-1 dyes were diluted to a final concentration of 2.5 µg/ml. The cells were incubated for 15 min at 21‒23°C in the dark. After incubation, cells were washed with PBS, and resuspended in 500 µl PBS. Flow cytometry analysis was performed immediately. The solutions were divided by NovoCyte 1000 and visualized by NovoExpress (ACEA biosciences).

### Statistical analysis

Student’s *t*-test was used to assess significant differences among treatment groups compared to untreated group using Excel. The criterion for statistical significance was set at *p* < 0.01. Values of *p* < 0.01 were considered statistically significant.

## Results and discussion

### Liquid chromatography (LC)- mass spectrometry (MS) analysis of the methanolic extract

In the present study, a total of 32 compounds were chromatographed in the methanolic extract of *P. elegans* by LC-MS (Figure S1, supplementary material). The retention time and mass spectrum characteristics of 28 identified and 4 unidentified compounds are systematically listed in Table 1. Moreover, the MS/MS fragmentation of unidentified compounds are summarized in Figure S2 (Supplementary material). The flavan-3-ols (e.g. catechins and their derivatives) were found as the dominating flavonoid polyphenols in the extract. Specifically, (-)-epicatechin and epicatechin-epicatechin (Dimer; proanthocyanidin) are identified from singly charged negative ions with a [M − H]^−^ at m/z 289.5 and 577.8, respectively (Figures S3 and S4, supplementary material). The retro-Diels-Alder (RDA) fragmentation of the epicatechin dimers produced typically [M − H − 152]^−^ product ions at m/z 425.7. The m/z of 407.6 [M − H − 152 − 18]^−^ resulted from the elimination of water molecule from m/z 425.7. The [M − H − 288] − ions at m/z 289.5 result from the loss of an epicatechin molecule [23]. With the help of selective multiple reaction monitoring (MRM) transitions, several non-flavonoid polyphenols, comprising of hydroxybenzoic acids, such as gallic, protocatechuic, 4-hydroxybenzoic, syringic, vanillic, and salicylic, and hydroxycinnamic acids, such as chlorogenic, caffeic, p-coumaric, and ferulic acids were identified (Table 1).

**Table 1.** Identification of major polyphenols in the methanolic extract of *P. elegans by* Liquid chromatography (LC)-electrospray ionization (ESI) -tandem mass spectrometric (MS/MS), and LC-ESI-MS/MS-multiple reaction monitoring (MRM) analysis.

S/No	ID	RT min	Exact Mass	MS *m/z*	MS/MS ions *m/z*	Identification method
1	Quinic acid	2.56	192.06	191	85	*b*
2	Gallic acid	5.16	170.02	169	125	*a*
3	Homogentisic acid (2,5-dihydroxyphenylacetic acid)	6.62	168.04	167.1	123, 122	*b*
4	Protocatechuic (3, 4-dihydroxybenzoic) acid	8.06	154.03	153	109, 91	*a*
5	3-O-Caffeoylquinic (chlorogenic) acid	9.37	354.1	353	191	*a*
6	Epigallocatechin or Gallocatechin	10.06	306.07	305	125	*b*
7	Epicatechin-Epicatechin (Dimer; Proanthocyanidin)	10.62	578.14	577.8		*c*
8	4-Hydroxybenzoic acid	11.25	138.07	137	93	*a*
9	(-)-Epicatechin	11.61	290.08	289.1	203, 245	*a*
10	Syringic acid	11.93	198.05	197	182	*a*
11	Vanillic acid	11.98	168.04	167.1	152, 108	*a*
12	Caffeic acid	12.05	180.04	179.1	135, 134	*a*
13	Unidentified	12.96	-	497.8	451.6, 420.6, 376.4, 290.3, 225.4	-
14	Quercetin-3-O-rutinoside (Rutin)	14.03	610.15	609	301	*a*
15	Epicatechin derivative	14.9	-	451.7	312.3, 297.1, 298.1, 167.1, 138.3, 123.2	*c*
16	p-Coumaric acid	15.74	164.05	163.1	119	*a*
17	Unidentified	15.77	-	724.1	679.0, 349.4, 311.5	-
18	Ferulic acid	16.53	194.06	193.1	134, 179, 149	*a*
19	Apigenin-hexose	16.88	432.11	431	269	*b*
20	Unidentified	17.88	-	469.7	452.4, 364.4, 348.4, 241.3, 121.1	-
21	Naringenin-hexose	18.14	434.12	433	271	*b*
22	Luteolin-hexose	18.4	448.1	447	285	*b*
23	Quercetin-hexose	18.68	464.1	463	301	*b*
24	Unidentified	18.77	-	187.3	143.2, 119.1	-
25	Rosmarinic acid	19.1	360.08	359	161	*b*
26	Salicylic acid	22.39	138.03	137	65	*a*
27	Luteolin	23.8	286.05	285	175, 199, 133	*a*
28	Quercetin	24.31	302.05	301	179	*a*
29	3,3ʹ-di-O-methyl ellagic acid	26.89	330.04	329.7	330.3, 212.2, 171.2, 139.3, 99.2	*c*
30	Apigenin	27.48	270.05	269	151	*a*
31	Naringenin	27.77	272.07	271	177	*a*
32	Isorhamnetin	28.67	316.06	315	300	*a*

RT: Retention time; *^a^* Identified using MRM and RT of authentic standards, *^b^* tentatively identified using reference MRM, and ^c^ tentatively identified using reference MS and MS/MS.

### Cell viability assay

**Figure 1.**
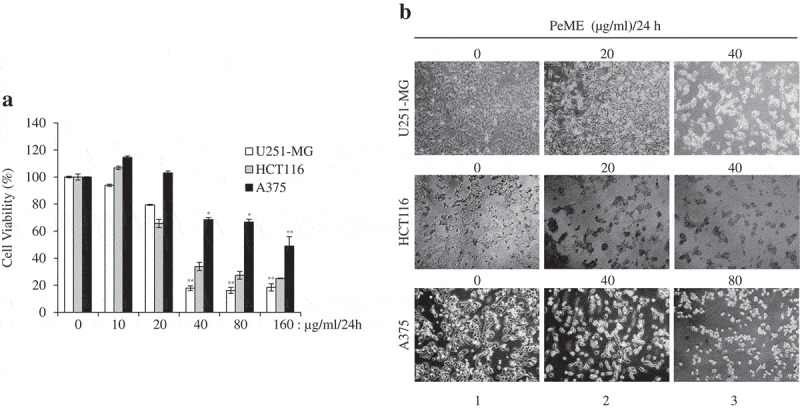
Cytotoxic effects of *P. elegans* methanolic extract (PeME)-treated glioblastoma, colon, and melanoma cancer cells. Cells were incubated for 24 h with various concentrations of PeME (0, 10, 20, 40, 80 and 160 μg/ml). (a). Cell viability was determined using WST-1 assay. Experiments were done in triplicates (n = 3) and the results were statistically significant at *P < 0.05, **P < 0.01. (**b)**. PeME treatment changed the cell morphology and inhibited the proliferation rate of glioblastoma, colon, and melanoma cancer cells. Cells were exposed to PeME at 0, 20, 40, and 80 µg/ml concentrations respectively. The cellular morphological changes were examined at 24 h of treatment and imaged under an inverted phase-contrast microscope (100x magnification).

Firstly, to determine the method of action of the plant extract on cancer cells, cell viability assay was performed with PeME in U251-MG, HCT116, and A375 cells. PeME treatment was done at various concentrations (0, 10, 20, 40, 80, and 160 µg/ml) for 24 h. Our findings revealed that cell growth was significantly suppressed in a concentration-dependent manner. Interestingly, it was observed that U251-MG and HCT116 cells were more sensitive toward PeME treatment than A375 cells. The IC50 values of PeME treated U251-MG, HCT116, and A375 cells were 28.275, 32.396, and 117.2 µg/ml respectively (Figure 1(a)).

### Morphological observation

The effect of PeME treatment on cell morphology in all three cell lines was studied. PeME treatment was done at various concentrations (0, 20, 40, 80 µg/ml) for 24 h. As shown in Figure 1(b), PeME-treated cells in a dose-dependent manner showed morphological features seen in the process of apoptosis (Lane 2–3), and not in the control (Lane 1). Treated cells clearly showed changes in their cellular morphology, cell shrinkage, and increased number of floating and dead cells. Proliferation rate in high concentration (40 and 80 µg/ml) of PeME was significantly reduced in all three cells. Subsequently, 40 µg/ml treatment of PeME in U251-MG and HCT116 cells showed that most of the cells were detached from the plate surface and floated in the media.

### In vitro scratch motility assay

**Figure 2.**
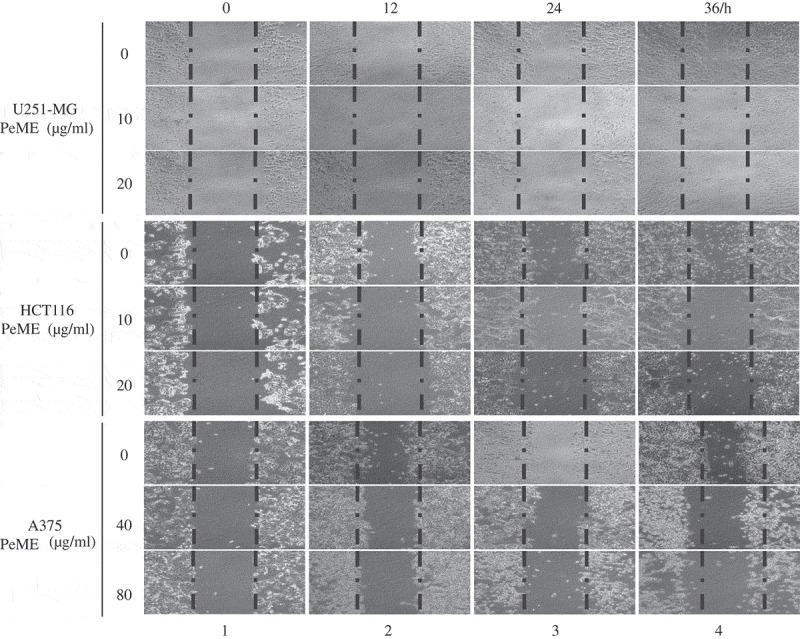
*P. elegans* methanolic extract (PeME) treatment showed the anti-metastatic effect and reduced the cell migration rate of the U251-MG, HCT116, and A375 cells. Wound closure ability of control and treated U251-MG (10 and 20 µg/ml), HCT116 (10 and 20 µg/ml), and A375 cells (40 and 80 µg/ml) were shown. The image of wounded wells was captured with the help of an inverted phase-contrast microscope at 0, 12, 24, 36 h (100x magnification).

Tumor metastasis is a major contributor to the death of cancer patients [24]. To determine whether PeME treatment induces anti-metastasis effect, *in vitro* scratch motility assay was performed. PeME was treated at various concentrations (0, 10, 20, 40, 80 µg/ml) for 0‒36 h. All three cells in the control wells migrated from the edges of the wound toward the centre of the scratched areas (Figure 2(a–c), lane 4, the uppermost). However, it had been observed that the cell migration rate was significantly reduced in PeME-treated wells, in a time-dependent manner (Figure 2(a–c), lane 2‒4, middle and bottom). Finally, these findings suggested that PeME showed potent cytotoxic, anti-metastatic, and anti-proliferative activity on cancer cells.

### Nuclear morphology study by 4′,6-diamidino-2-phenylindole (DAPI) staining

**Figure 3.**
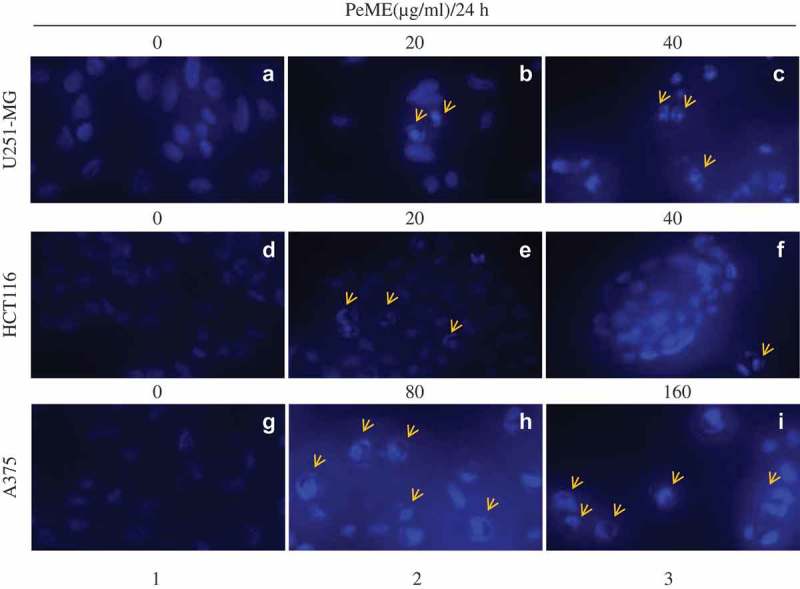
Apoptotic features in *P. elegans* methanolic extract (PeME) treated glioblastoma, colon, and melanoma cancer cells. (a–c) showed control, 20 and 40 µg/ml treated U251-MG and (d–f) showed control, 20 and 40 µg/ml treated HCT116 incubated for 24 h. Changes in the morphology of the cancer cells were observed under fluorescence microscope (200x magnification).

To examine the morphological change of the cells after PeME treatment, DNA-specific fluorescent dye DAPI was used to stain cell nuclei. To do this, PeME was treated at various concentrations (0, 20, 40, 80, and 160 µg/ml) for 24 h. As shown in Figure 3, all three cells showed normal intact nuclei and less bright blue fluorescence in the centre (a/d/g). In contrast, PeME-treated cells (b-c/e-f/h-I, arrow) showed bright blue fluorescent dots in the centre as well as condensed and fragmented nuclei. Moreover, at higher concentrations (c/f/i, arrow), cells with distorted nuclei were increased in number in all the treatments. Overall, our findings suggest that PeME treatment induces apoptotic features such as damaged and condensed nuclei in a dose-dependent manner.

### Annexin V staining for apoptosis detection

**Figure 4.**
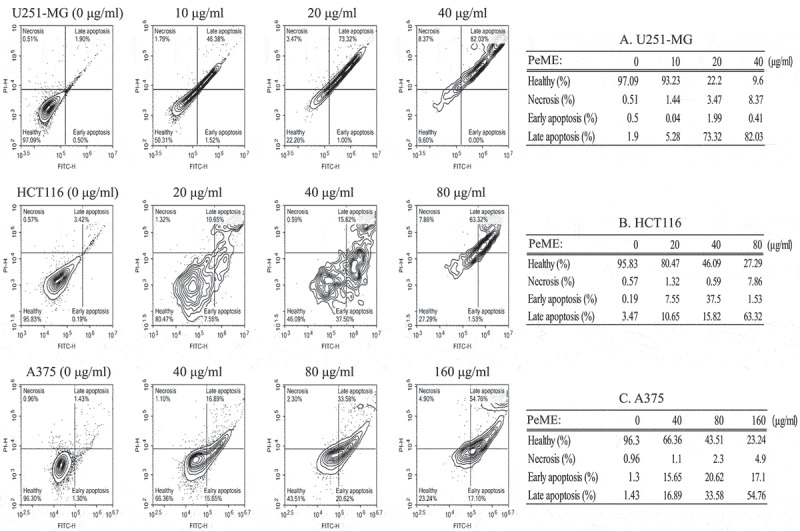
Flow cytometry analysis of U251-MG, HCT116, and A375 cell lines after *P. elegans* methanolic extract (PeME) treatment for 24 h. All cells were labeled with annexin V-FITC and PI. U251-MG cells (a) treated at 0,10, 20, and 40 µg/ml concentration of PeME, HCT116 cells (b) were treated for 0, 20, 40, and 80 µg/ml. (b), A375 cells (c) were treated 0, 40, 80, and 160 µg/ml. FACs analysis was performed and analyzed. Data presented are representative of three experiments.

Induction of apoptosis is one of the most important markers of cytotoxic antitumor agents. It has been shown that some natural compounds including plants induce apoptotic pathways that are blocked in cancer cells [3]. The apoptotic event following treatment of PeME on three cell lines was investigated. After treatment with concentrations of PeME for 24 h, cells were labeled with annexin V-FITC and PI and flow cytometry analysis was performed. These findings suggested that PeME treated U251-MG and HCT116 (20 and 40 µg/ml) cells and A375 (80 and 160 µg/ml) cells exhibited a change in nuclear morphology such as condensed and fragmented nuclei, and damaged and shrunk nuclear membrane as compared to control cells (data not shown). Apoptotic cell number was found to be significantly higher in a directly proportional dose-dependent manner in all three cell types following treatment. As shown in Figure 4, apoptotic cell population was significantly increased in a dose-dependent manner in PeME treated cancer cells. U251-MG cells (Table A) were more sensitive to PeME treatment (Figure 4, upper). These findings were already reaching 75% of apoptotic deaths at 20 µg/ml treated cells, compared to HCT116 (Table B: 18.2% death at 20 µg/ml) and A375 (Table C: 32.54% death at 40 µg/ml) cells. Early apoptosis occurred in the order of HCT116 (Table B: 37.5% death at 40 µg/ml), A375 (Table C: 20.62% death at 80 µg/ml), and U251-MG (Table A: 1.99% at 20 µg/ml). On the other hand, late apoptosis occurred in the order of U251-MG (Table A: 82.03% death at 40 µg/ml), HCT116 (Table B: 63.32% death at 80 µg/ml), and A375 (Table C: 54.76% death at 160 µg/ml). Early apoptosis occurred in the order of HCT116 (Table B: 37.5% death at 40 µg/ml), A375 (Table C: 20.62% death at 80 µg/ml), and U251-MG (Table A: 1.99% death at 20 µg/ml). On the other hand, late apoptosis occurred in the order of U251-MG (Table A: 82.03% death at 40 µg/ml), HCT116 (Table B: 63.32% death at 80 µg/ml), and A375 (Table C: 54.76% death at 160 µg/ml). These differences appear to be related to cancer cell type-specific effects.

### P. elegans methanolic extract (PeME)-induced cell death occurred via caspase-3/-9 and Mu-2-related death-inducing gene (MuD) activation

**Figure 5.**
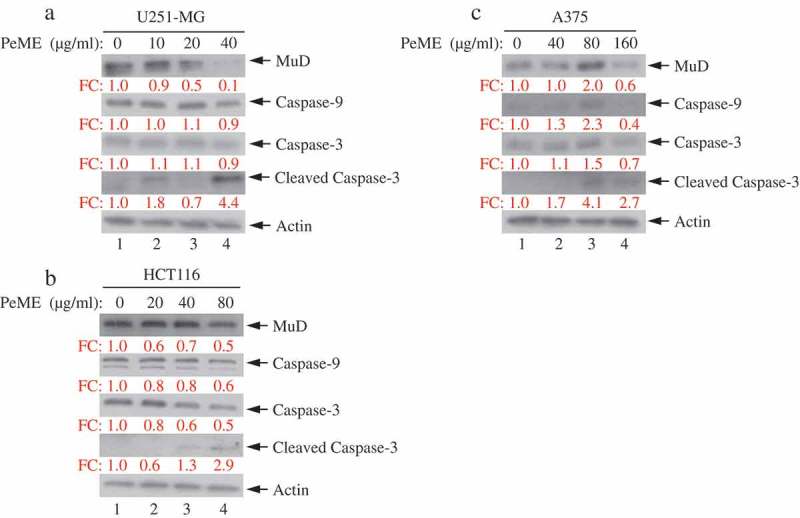
Analysis of caspase and MuD activation in U251-MG, A375, and HCT116 cell lines. Cells were treated with various concentrations of PeME for 24 h; U251-MG were treated with 0, 10, 20 and 40 µg/ml (a), HCT116 cells were treated with 0, 10, 20, 40 and 80 µg/ml (b), A375 cell were treated with 0, 40, 80 and 160 µg/ml (c). The lysates were analyzed by immunoblotting using anti-caspase-3/-9 and MuD MAb. Actin was used for loading control.

Next, the dynamics of key components involved in the extrinsic apoptosis pathway was examined. Various concentrations of PeME were treated in each cell for 24 h and then western blot was performed using specific target Abs. As shown in Figure 5(a–c), it was observed that PeME treatment induces proteolytic cleavage of caspase-3/-9 molecules at high concentration in each cell. Previously, it has been suggested that MuD may be a novel caspase-3 substrate in response to TRAIL stimulation in Jurkat T cells [18,25]. Thus, it was examined whether MuD expression in these cells could be modulated by PeME. As shown in Figure 5(a–c), constitutive expression of MuD protein was detected (lane 1). Interestingly, the amount of MuD protein began to decrease at low concentrations (lane 2‒3), reaching maximal reduction at high concentrations (lane 4). These results demonstrate that MuD and caspase-3/-9 activation were involved in PeME induced cell death.

### Measurement of mitochondria membrane potential (MMP)

**Figure 6.**
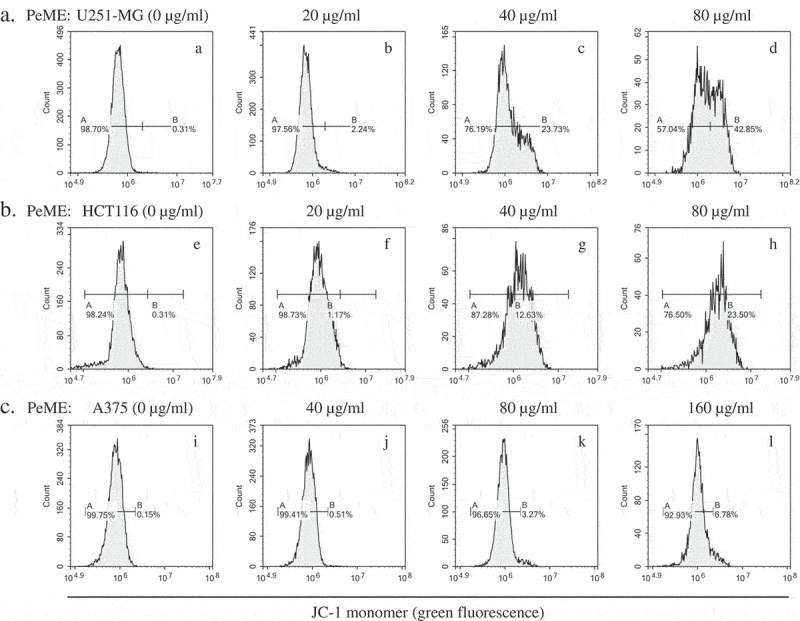
*P. elegans* methanolic extract (PeME) alleviated mitochondrial membrane potential (MMP). PeME induced a significant reduction in MMP in three cells. a-d showed control, 20, 40, and 80 µg/ml treated U251-MG; e-h showed control, 20, 40, and 80 µg/ml treated HCT116; **i-l** showed control, 40, 80, and 160 µg/ml treated A375 cells. Histogram profiles of JC-1 monomer (green fluorescence) were detected using flow cytometry. Peaks of treated concentration were shifted toward the right in the three treated cells compared to control, indicating a reduction of MMP.

Mitochondrial dysfunction has been shown to participate in the induction of apoptosis [26]. Flow cytometric JC-1 staining was done to determine whether PeME treatment triggers apoptotic pathway by mitochondrial dysfunction. As shown in Figure 6, we observed right-shifted green fluorescence profiles in a dose-dependent manner in PeME-treated cells was observed. Furthermore, through checking the MMP after PeME treatment, we observed a significant reduction in MMP in a dose-dependent manner. It is indicated that PeME triggers mitochondrial dysregulation, leading to apoptotic signaling pathway.

In spite of improved surgical techniques and advanced radio/chemotherapy, the survival rate and life expectancy of cancer patients have not been extended because of side effects and resistance to anti-cancer drugs. Therefore, the availability of natural products with higher effectiveness and lower side effects is desired [4]. Medicinal herbs are important for cancer treatment as they possess multiple chemical compounds, which can be used to discover new active materials against cancer [27]. *P. elegans* (Lour.) Desv. (taxon name) has an application in traditional medicinal in southeastern Asia. The roots and leaves are particularly used in folk medicine [10,12]. It was displayed in only one species of the *Phyllodium* genus that a substance isolated from *P. pulchellum* extracts inhibited the proliferation of the human hepatocyte carcinoma cell lines, *in vitro* [28].

Recently Li *et al* reported for the first time that three triterpenoids (lupenone, lupeol, and botulin) are chemical constituents of *P. elegans* extracts [22]. However, they did not provide additional information on the function of the triterpenoids in *P. elegans* extracts. Those triterpenoids have a wide range of pharmacological effects including anti-inflammatory, anti-diabetic and anti-cancer effects, and stimulation of various immune functions [29]. For example, lupeol has been discovered as an active inhibitor of some cancers including prostate and skin cancers via downregulation of tumor necrosis factor (TNF)-α and VEGF-R2 [30–32]. Moreover, lupenone has been known to stimulate melanogenesis through the inhibition of Erk1/2 activation [33].

Since then, there has been no report on the biological activity of *P. elegans* extracts. Therefore, this study to investigate whether PeME has anti-cancer activity against human astroglioma cells (U251-MG), human colorectal carcinoma cells (HCT116), and human malignant melanoma cells (A375) was undertaken. These findings revealed that PeME displayed cytotoxicity against all three cell lines. Clear apoptotic changes in these cells were observed after treatment. Cell migration and invasion rate of the cancer cells by *in vitro* scratch invasion assay were significantly reduced. JC-1 staining analysis by flow cytometry showed that PeME depolarized the MMP. Thus, these results enabled us to report that PeME has anti-tumoral potential against cancer cells by depolarization of MMP and activation of apoptotic signaling through the activation of caspase-3/-9 and MuD.

MuD, the novel anti-apoptotic protein is expressed and has a function in human glioblastoma cells [18]. The function of this novel protein is inhibited upon TNF-related apoptosis-inducing ligand (TRAIL) stimulation in astroglioma cells [18]. In this study, PeME induced activation of caspase-3/-9 and decreased MuD protein expression in a dose-dependent manner, suggesting that MuD is involved in PeME’s activities to suppress cell growth was observed. The novelty of this work is the very fact that this plant extract is seldom worked on and this is the first report on working out the anticancer activity and its effect on MuD. Thus, this naïve extract is not yet studied and it adds novel value toward natural product research. This research is expected to trigger off sensitization for application toward versatile anticancer natural remedies. More detailed future studies unraveling the exact mechanism and fundamental level of inhibitory mechanisms behind the anticancer activity of *Phyllodium elegans* extracts could result in meaningful and fruitful applications and natural remedies for cancer.

## Conclusion

In conclusion, PeME anti-cancer activity against U251-MG, HCT116, and A375 cells in a time-/dose-dependent manner has been demonstrated. The U251-MG cells were most sensitive to PeME treatment compared to the other cells. The data also confirmed the apoptotic and anti-metastatic properties of PeME. This study, for the first time, confirmed that PeME triggered apoptotic cell death through significant reduction in MMP in cancer cells. This naïve extract is not yet studied, so it has novel value for natural product research.
