# Supplementary material for: Cytotoxic and apoptotic potential of Phyllodium elegans extracts on human cancer cell lines
Source: Bioengineered. 2019 Oct 29;10(1):501–12. doi: 10.1080/21655979.2019.1682110 (PMC6844383; doi:10.1080/21655979.2019.1682110)
Supplement: Supplemental Material [file kbie-10-01-1682110-s001.docx]

**Supplementary files**

**Cytotoxic and apoptotic potential of *Phyllodium elegans* extracts on human cancer cell lines**
Seunghwa Jung^1^, Juhyun Shin^1^, Jeongheon, Oh^1^, Gansukh Enkhtaivan^2^, Sang Woo Lee^3^, Kongmany Sydara^4^, Judy Gopal^2^, Ramesh Kumar Saini^5^, Young Soo-Keum^5^, and Jae-Wook Oh^1,*^

^1^Department of Stem Cell and Regenerative Biotechnology, KIT, ^2^Department of Bioresource and Food Science, Konkuk University, Seoul, 05029 Korea

^3^International Biological Material Research Center, Korea Research Institute of Bioscience and Biotechnology, Daejeon, 34141, Republic of Korea

^4^Ministry of Health, Institute of Traditional Medicine, Vientiane 116, Lao PDR

^5^Department of Crop Science, Konkuk University, Seoul, 05029 Korea

^*^Corresponding author:

Jae-Wook Oh, PhD

Department of Stem Cell and Regenerative Biotechnology,

KIT, Konkuk University,

120 Neungdong-ro, Gwangjin-gu, Seoul 05029, Republic of Korea

Tel: +82-2-2049-6271

E-mail: [ohjw@konkuk.ac.kr](mailto:ohjw@konkuk.ac.kr)

**Table S1.** Optimized values of declustering potential (DP), collision energy (CE), entrance potential (EP), cell exit potential (CXP) of MRM transition used for the liquid chromatography (LC)-multiple reaction monitoring (MRM)-mass spectrometry (MS) quantification of major polyphenols from the methanolic extract *P. elegans*.

| S/No | ID | MRM transitions (Q1/Q3) | DP | CE | EP | CEP |
| --- | --- | --- | --- | --- | --- | --- |
| 1 | Quinic acid | 191/85 | -40 | -20 | -5 | -10 |
| 2 | Gallic acid | 169/125 | -40 | -20 | -5 | -10 |
| 3 | Melanic (Homogentisic) acid | 167.1/123, 167.1/122 | -40 | -20 | -5 | -10 |
| 4 | Protocatechuic (3, 4-dihydroxybenzoic) acid | 153/109, 153/91 | -24 | -15 | -5 | -10 |
| 5 | 3-O-caffeoylquinic (chlorogenic) acid | 353/191 | -24 | -15 | -5 | -10 |
| 6 | Epigallocatechin or Gallocatechin | 305/125 | -50 | -40 | -5 | -15 |
| 7 | 4-Hydroxybenzoic acid | 137/93 | -24 | -15 | -5 | -10 |
| 8 | (-)-Epicatechin | 289.1/203, 289.1/245, 289.1/203 | -50 | -40 | -5 | -15 |
| 9 | Syringic acid | 197/182 | -40 | -20 | -5 | -10 |
| 10 | Vanillic acid | 167.1/152, 167/108 | -28 | -10 | -5 | -10 |
| 11 | Caffeic acid | 179.1/135, 179/134 | -36 | -20 | -5 | -10 |
| 12 | Quercetin-3-O-rutinoside (Rutin) | 609/301 | -64 | -46 | -5 | -22 |
| 13 | p-Coumaric acid | 163.1/119 | -30 | -20 | -5 | -10 |
| 14 | Ferulic acid | 193.1/134, 193.1/179, 193.1/149 | -30 | -20 | -5 | -10 |
| 15 | Apigenin-hexose | 431/269 | -70 | -45 | -7 | -15 |
| 16 | Naringenin-hexose | 433/271 | -64 | -46 | -5 | -22 |
| 17 | Luteolin-hexose | 447/285 | -70 | -40 | -5 | -15 |
| 18 | Quercetin-hexose | 463/301 | -64 | -46 | -5 | -22 |
| 19 | Rosmarinic acid | 359/161 | -40 | -20 | -5 | -10 |
| 20 | Salicylic acid | 137/65 | -24 | -15 | -5 | -10 |
| 21 | Luteolin | 285/175, 285/133, 285/199 | -58 | -30 | -5 | -15 |
| 22 | Quercetin | 301/179 | -45 | -30 | -5 | -22 |
| 23 | Apigenin | 269/151 | -60 | -35 | -7 | -15 |
| 24 | Naringenin | 271/177 | -45 | -30 | -5 | -22 |
| 25 | Isorhamnetin | 315/300 | -40 | -20 | -5 | -10 |

**Figure S1.** The total ion chromatogram (TIC) of methanolic extract of *P. elegans* obtained by liquid chromatography (LC)-electrospray ionization (ESI^-^) -tandem mass spectrometry

**Figure S2.** The MS/MS fragmentation pattern of unidentified compounds

**Figure S3.** The MS/MS fragmentation pattern of epicatechin-epicatechin (Dimer; Proanthocyanidin; Peak number 7)

**Figure S4.** The MS/MS fragmentation pattern of (-)-epicatechin-Epicatechin (Peak number 9)
